# Unveiling the role of NAD glycohydrolase CD38 in aging and age-related diseases: insights from bibliometric analysis and comprehensive review

**DOI:** 10.3389/fimmu.2025.1579924

**Published:** 2025-06-02

**Authors:** Xianghui Zhao, Peiying Lv, Zixing Cai, Jun Dong, Wenxiang Chen, Liang Sun, Ruiyue Yang

**Affiliations:** The Key Laboratory of Geriatrics, Beijing Institute of Geriatrics, Institute of Geriatric Medicine, Chinese Academy of Medical Sciences, Beijing Hospital/National Center of Gerontology of National Health Commission, Beijing, China

**Keywords:** CD38, aging, bibliometric analysis, metabolic diseases, tumors

## Abstract

**Background:**

CD38, a glycoprotein with a single transmembrane structure, is extensively found in erythrocytes, immune cells, and endothelial cells. Primarily located on cell membranes, it plays a critical role in metabolizing nicotinamide adenine dinucleotide (NAD), thereby maintaining NAD homeostasis *in vivo*. As a vital coenzyme, NAD is involved in numerous biological processes, including energy metabolism, apoptosis, and DNA repair. CD38, as a major NAD-depleting enzyme, is pivotal in regulating intracellular NAD levels and various physiological processes. Given its significance, understanding the function of CD38 and its implications in aging and age-related diseases is crucial for elucidating disease pathogenesis and developing therapeutic strategies.

**Methods:**

This study conducted a bibliometric analysis to explore recent research trends and advancements in the field of CD38. Research articles were retrieved from the Web of Science database, followed by a bibliometric assessment using CiteSpace and VOSviewer to visualize key publication trends, contributions by countries and institutions, and keyword distributions. Based on the bibliometric analysis, key insights were synthesized to elucidate the role of CD38 in aging and age-related diseases, its underlying mechanisms, and its applications in clinical evaluation, detection methods, interventions, and therapeutic targets.

**Results:**

The bibliometric analysis revealed an exponential increase in the number of published articles over time, with the United States and China emerging as the leading research hubs. The predominant keywords included ‘CD38’ and ‘blood-related disorders’. Furthermore, key findings highlighted the critical role of CD38 in aging and age-related diseases, emphasizing its mechanisms in NAD metabolism and its potential as a therapeutic target. Moreover, current applications of CD38 in clinical evaluation and detection methods were discussed, showcasing its growing importance in biomedical research.

**Conclusion:**

This study underscores the growing interest in CD38 research, particularly its role in aging and age-related diseases. The findings highlight the significance of CD38 in maintaining NAD homeostasis and its potential as a therapeutic target. The exponential growth in publications and the dominance of the United States and China in this field reflect the global importance of CD38 research. Future studies should further explore the mechanistic insights and clinical applications of CD38 to advance therapeutic strategies for age-related diseases.

## 1 Introduction

The global population is aging at an unprecedented rate, bringing age-related diseases to the forefront of public health challenges (1). Conditions such as cancers, metabolic dysfunctions and other age-related diseases, including neurodegenerative disorders are increasingly prevalent, imposing significant burdens on healthcare systems worldwide (2, 3). Aging is a complex biological process influenced by a myriad of factors, notably the decline of essential molecules like nicotinamide adenine dinucleotide (NAD). Emerging research has highlighted the enzyme CD38, a critical regulator of NAD levels, as a pivotal player in the aging process and the pathogenesis of age-related diseases (4–6).

### 1.1 Discovery and cellular localization of CD38

CD38 is a single-chain transmembrane glycoprotein encoded by the CD38 gene on chromosome 4, consisting of 300 amino acids. Its structure includes a short 21-amino acid N-terminal tail and a 23-amino acid hydrophobic transmembrane domain, with a large extracellular C-terminal region comprising 256 amino acids (7). Structural studies have revealed a strong similarity between CD38 and the sea hare ADP-ribosyl cyclase (8). This structural homology underscores the evolutionary conservation of NAD-metabolizing enzymes across species, suggesting that CD38’s catalytic mechanism may share fundamental principles with these distantly related enzymes, thereby supporting its functional importance in mammals. Additionally, CD38 shares high homology with the NAD-specific hydrolase from rabbit eggs (7). The comparison to rabbit and sea hare enzymes highlights conserved functional domains critical for enzymatic activity, reinforcing the idea that CD38’s role in NAD metabolism is deeply rooted in evolutionary biology and not merely a species-specific adaptation.

Initially discovered as a T-cell surface marker (9, 10), CD38 is now recognized for its widespread expression in various cells, including erythrocytes, macrophages, dendritic cells, neutrophils, lymphocytes, endothelial cells, and precursor immune cells (11). Structurally, the majority of CD38 molecules function as type II transmembrane proteins with an extracellular catalytic site (10, 12). A smaller fraction exists as type III proteins with cytosolic catalytic domains, and soluble CD38 isoforms are found extracellularly (10, 13, 14). Intracellular localization to organelles such as the endoplasmic reticulum, mitochondria, and nuclear membrane has also been reported (10, 11), highlighting the complex subcellular distribution of CD38.

### 1.2 Enzymatic and receptor functions of CD38

CD38 exerts multiple enzymatic activities critical for cellular signaling and metabolism.

Primarily, CD38 acts as a NAD glycohydrolase, cleaving NAD to generate nicotinamide (NAM) and adenosine diphosphate ribose (ADPR). Additionally, it functions as an ADP-ribose cyclase, converting NAD into NAM and cyclic ADP-ribose (cADPR), a potent second messenger that activates ryanodine receptors on the endoplasmic reticulum to induce intracellular Ca²^+^ release (6, 15). Under acidic conditions, CD38 also catalyzes a base exchange reaction between NAD(P) and nicotinic acid (NA), producing NAM and nicotinic acid adenine dinucleotide (phosphate) (NAAD(P)) (16).

Beyond enzymatic function, CD38 functions as a cellular receptor. It interacts with CD31 to mediate immune cell adhesion and transmigration across endothelial barriers (17). In hematological malignancies, the CD38-CD31 axis may promote lymphocyte proliferation, particularly in chronic lymphocytic leukemia (18, 19).CD38 also contributes to innate immune responses, as CD38-deficient mice display impaired immune responses following bacterial infections (16). Furthermore, CD38 influences cytokines production, regulating IL-1, IL-6, IL-10, IFN-γ, and GM-CSF (20), although the extent to which these effects depend on its enzymatic function remains to be clarified.

### 1.3 CD38 as a mediator of aging and age-related diseases

Emerging evidence underscores the pivotal role of CD38 in aging biology (21–25). Declining NAD levels are now recognized as a major driver of physiological deterioration during aging and the development of age-related diseases (24, 25). Elevated CD38 activity has been correlated with age and pathological conditions. Cagatay et al. reported that CD38 enzymatic activity in cancer patients was two to three times higher than that in healthy controls (26), and Polzonetti found a significant positive correlation between CD38 activity and aging (27). Camacho et al. further demonstrated that increased CD38 expression leads to NAD+ depletion and mitochondrial dysfunction in aged mice via a SIRT3-dependent mechanism (28). Given its role in NAD metabolism, CD38 has emerged as a promising therapeutic target for various diseases, with strategies including cytotoxic antibodies, enzymatic inhibitors, and small-molecule antagonists showing therapeutic potential (14).

### 1.4 Knowledge gaps and research strategy

Despite extensive research, two major gaps remain (1): a lack of quantitative characterization of research trends across different disciplines, and (2) incomplete mechanistic understanding of CD38’s dual roles in health and disease. To address these gaps, we conducted a bibliometric analysis spanning 2004–April, 2025, incorporating temporal trend analysis, co-citation network modeling, and keyword burst detection. This multidimensional strategy bridges mechanistic insights with therapeutic development frameworks, offering a novel perspective on CD38’s role in aging biology and age-related pathologies.

## 2 Methods

This investigation employed a dual-modality analytical framework integrating scientometric topology mapping with systematic knowledge synthesis to delineate CD38’s functional spectrum in aging pathobiology. The experimental architecture was structured around three investigational axes:

1. Identification of specific age-related disease clusters associated with CD38 pathophysiology;
2. Mechanistic deconvolution of CD38-mediated aging processes;
3. Systematic evaluation of CD38 detection methodology evolution.

**Figure 1:**
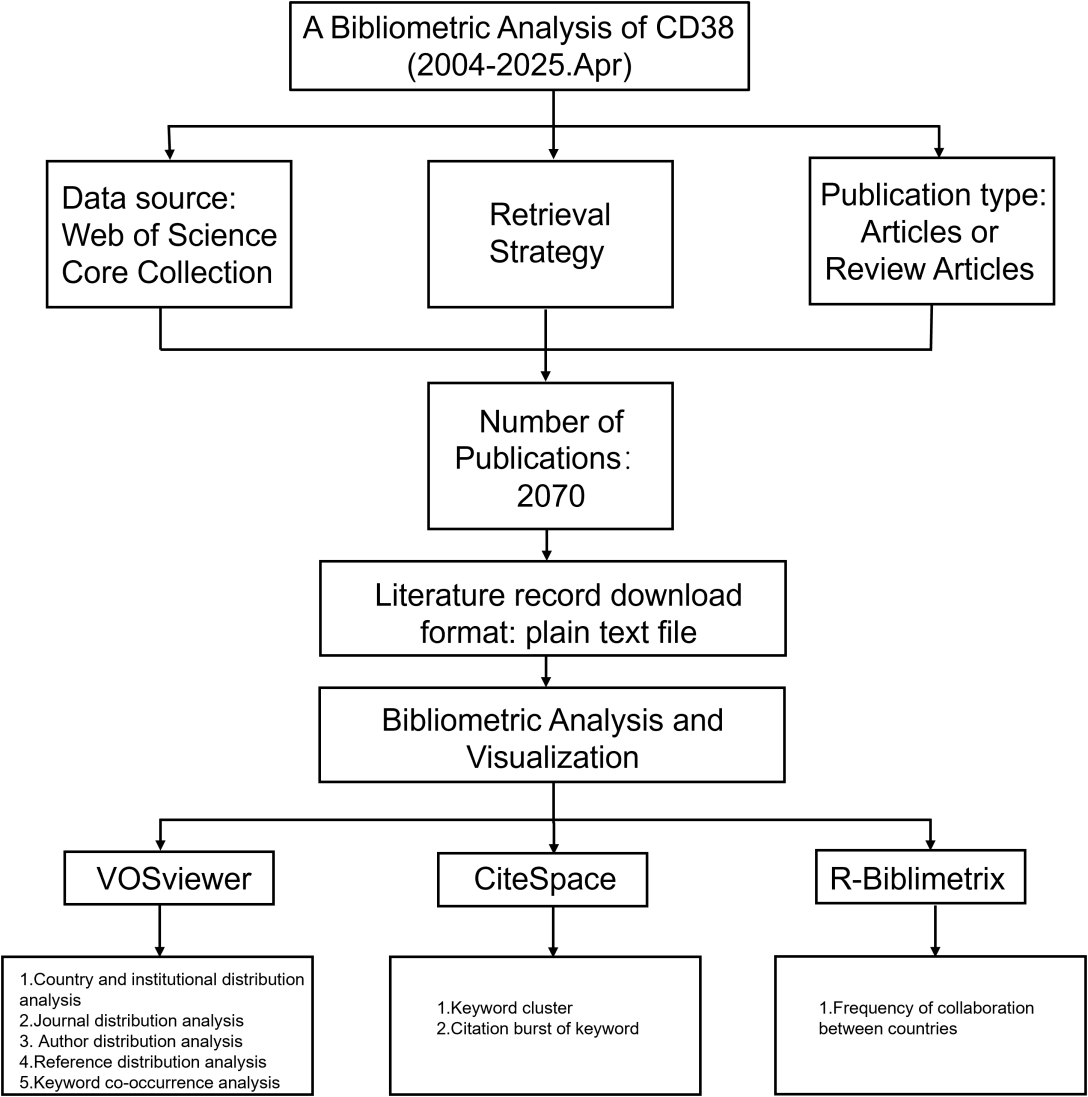
Flow chart of the study.

The knowledge discovery process commenced with computational literature interrogation of the Web of Science Core Collection on April 18, 2025, using predefined keywords. The search strategy included the following queries: TS=(CD38) AND ((TS=(Aging) OR TS=(Ageing)) OR (TS=(Age-related) AND TS=(Disease)) OR TS=(Atherosclerosis) OR TS=(Coronary Atherosclerotic Heart Disease) OR (TS=(Metabolic) AND TS=(Disease)) OR TS=(Cancer)).The search was restricted to original articles and review articles published between January 2004 and April 2025. In total, 2070 publications were identified. A detailed overview of the selection process is illustrated in **Figure 1**. The curated literature corpus underwent systematic analytical processing integrating validity-weighted methodological appraisal with machine learning-driven semantic topology mapping to resolve the predefined research inquiries.

## 3 Results

### 3.1 Bibliometric analysis

#### 3.1.1 Publication trend

**Figure 2:**
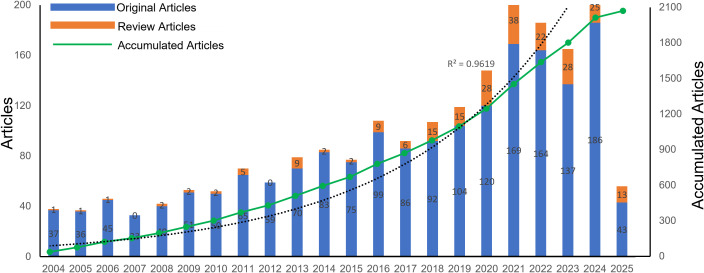
Number of publications per year and the cumulative number.

As of April 18, 2025, 2070 publications related to CD38 in aging and age-related diseases have been identified, comprising 226 review articles and 1,844 original articles. As illustrated in **Figure 2**, the overall number of publications has shown an upward trend over the past two decades, although the annual number of review articles significantly fluctuated. Before 2009, the growth in the number of original articles was relatively slow, with an average of 39.2 publications per year, and the annual output never exceeded 45 articles. However, starting in 2009, the publication rate markedly increased, reaching an average of 110.2 articles per year. The number of original articles peaked in 2024 with 211 publications. Although there was a slight decline from 2021 to 2024, the publication volume remained high. An exponential growth function was applied to assess the relationship between the cumulative number of articles and the publication year, demonstrating a strong fit to the data (R² = 0.9619). This indicates that CD38 has become an increasingly prominent research focus in the field of aging and age-related diseases. Furthermore, the number of review articles has also significantly increased since 2016, reflecting growing interest in this field of study.

#### 3.1.2 Country and institutional distribution analysis

These publications originated from 51 countries/regions and 109 institutions. The United States ranked first with 745 publications, followed by China (383), Italy (208), the United Kingdom (167) and Germany (157), with the remaining countries and regions each published fewer 70 articles (**Supplementary Figure S1**). TheVOSviewer network map visualized these countries and their collaboration strength, highlighting strong partnerships between China and the United States, the United States and Italy, and Germany and the United States (**Supplementary Figure S2A**). A time-overlay map revealed that recent contributions have come from China, Norway, Qatar, and Mexico (**Supplementary Figure S3A**). The top 19 institutions (**Supplementary Figure S4**) were led by Mayo Clinic (56 publications), followed by University of San Francisco (51), and the University of Texas MD Anderson Cancer Center (43). Institutional collaborations were visualized in VOSviewer, showing eight clusters, with the largest red cluster including Mayo Clinic, University of San Francisco, and Johns Hopkins (**Supplementary Figure S2B**). The time-overlay network map highlighted institutions like Ulm University and Dana-Farber Cancer Institute as early contributors, while newer institutions include Washington University and Huazhong University (**Supplementary Figure S3B**).

#### 3.1.3 Journal distribution analysis

Publications on CD38 in aging and age-related diseases were distributed across 89 journals. **Supplementary Table S1** lists the top 10 journals ranked by the number of publications, along with their most recent impact factors. “Frontiers in Immunology” published the most articles (69 articles), followed by “Plos One” (67 articles), “Blood” (42 articles), “Cancers” (39 articles), and “Aids” (30 articles). Seven of the top 10 journals were JCR Q1, with Sweden leading with four journals. The journals’ impact factors in 2025 ranged from 2.9 to 21, with “Blood” having the highest (IF = 21). Analysis of co-cited journals is presented in **Supplementary Table S2**. Co-cited journals, with over 1,000 citations each, included “Blood” (8515 citations), the “Journal of Immunology” (2,293 citations), and “New England Journal of Medicine” (2,250 citations). VOSviewer visualized the journal and co-citation networks, showing strong citation relationships(**Supplementary Figure S5**).

#### 3.1.4 Author distribution analysis

A total of 15,707 authors contributed to CD38 research. **Supplementary Table S3** highlights the authors with the highest number of publications and citations in this field. Fabio Malavasi from the University of Turin ranked first in publications (23 articles) and citations (255 citations), followed by Eduardo Nunes Chini from the Mayo Clinic (16 articles). The most cited author after Malavasi was Rajendra N. Damle (236 citations). These data underscore the significant contributions of these researchers to studying the role of CD38 in the context of aging and age-related diseases.

#### 3.1.5 Reference distribution analysis

A total of 70,715 references were cited. **Supplementary Table S4** lists the top 10 most frequently cited studies, all of which have been cited over 90 times. The most frequently cited study, “Ig V gene mutation status and CD38 expression as novel prognostic indicators in chronic lymphocytic leukemia” (1999) by Damle et al., received 190 citations. The main finding of this study is that CD38 expression serves as a valuable adjunct in predicting the clinical course of individual B-CLL cases. The second most cited study, “Evolution and function of the ADP ribosyl cyclase/CD38 gene family in physiology and pathology” by Malavasi et al., received 135 citations. This study reviews the potential roles of CD38 in diagnosis, prognosis, and therapeutic strategies from the perspective of pathological mechanisms. These studies provided strong evidence for the prognostic significance of CD38 and serving as foundational contributions to the field.

#### 3.1.6 Keyword co-occurrence cluster analysis

**Figure 3:**
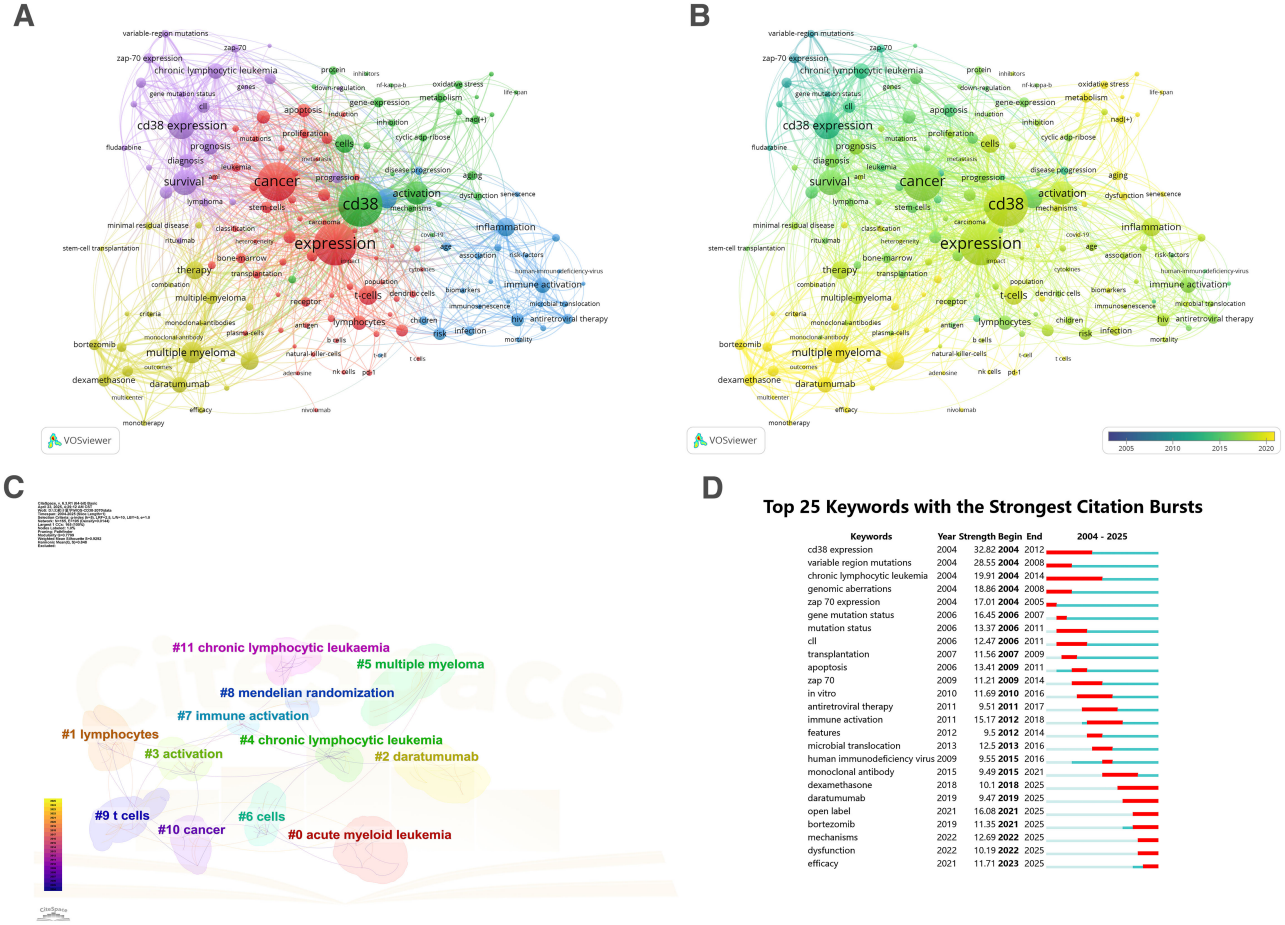
Research keywords on CD38 in aging and age-related diseases. **(A)** Keyword clusters color-coded by research themes; **(B)** Temporal evolution of research topics; **(C)** Refined keyword clusters identified by CiteSpace; **(D)** Top 25 keywords with strongest citation bursts.

Keywords provide a concise and precise summary of research themes, and co-occurrence analysis of keywords offers a rapid way to identify research hotspots in the field of CD38 in aging and age-related diseases. Using VOSviewer and CiteSpace, we conducted a co-occurrence analysis of all 161 keywords. are listed in **Supplementary Table S5**. These keywords were grouped into five clusters, each presented in a different color (**Figure 3A**) and indicating distinct research directions. Keywords in the red cluster included cancer, carcinoma, leukemia, acute myeloid leukemia, and bone-marrow; keywords in the green cluster included CD38, NAD, activation, oxidative stress, metabolism, and aging; keywords in the blue cluster included inflammation, risk-factor, biomarkers, immunosenescence and children; keywords in the yellow cluster included multiple myeloma, daratumumab, dexamethasone, monoclonal-antibody and therapy; keywords in the purple cluster included CD38 expression, prognosis, diagnosis, chronic lymphocytic leukemia and ZAP-70. A time-overlap visualization (**Figure 3B**) revealed that recent studies have focused on emerging topics, such as aging, biomarker, therapy, and tumor microenvironment.

Using the log-likelihood ratio-based algorithm in CiteSpace, keyword clustering was refined. The modularity value (Q = 0.848, where Q > 0.8 indicates reasonable clustering) confirmed the validity of the analysis. **Figure 3C** shows five main clusters: #0acute myeloid leukemia, #1lymphocytes, #2daratumumab, #3activation, #4chronic lymphocytic leukemia, #5multiple mteloma, #6cells, #7immune activation, #8mendelian randomization, #9t cells, #10cancer, #11chronic lymphocytic leukemia. These clusters reflect research themes including acute and chronic hematological diseases, tumors, immune activation, and gene expression. **Figure 3D** lists the top 25 keywords with the strongest citation bursts lasting at least one year. CD38 expression received sustained attention from 2004 to 2012, while recent bursts have focused on keywords such as daratumumab, bortezomib, mechanisms, dysfunction, and efficacy, indicating future research trends.

**Figure 4:**
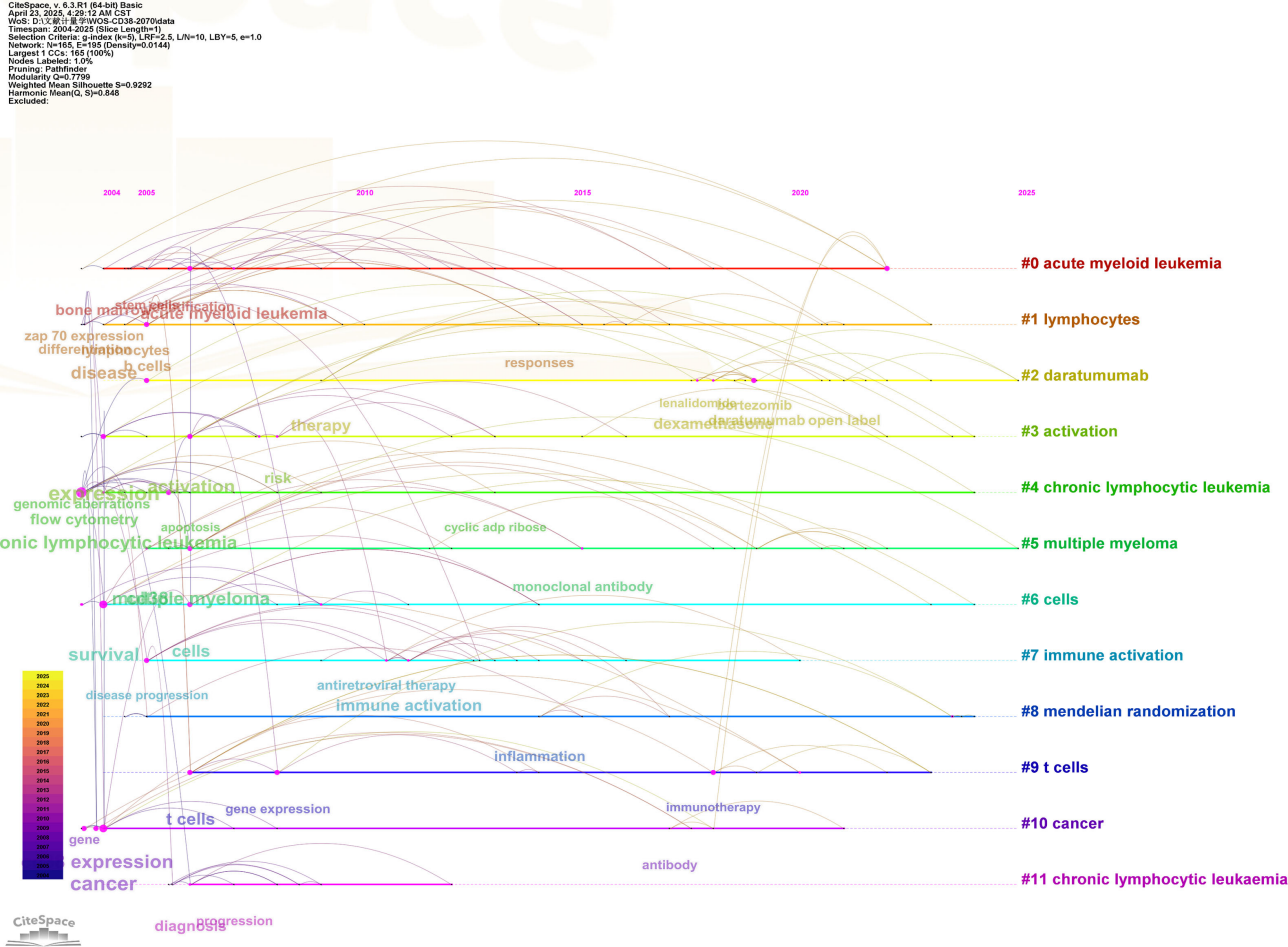
CiteSpace visualization map of timeline viewer related to CD38 in aging and age-related diseases.

A keyword timeline clustering was conducted using CiteSpace to explore the dynamic evolution of research hotspots (**Figure 4**). Between 2004 and 2005, studies have primarily focused on CD38 expression, chronic lymphocytic leukemia, acute lymphocytic leukemia and flow cytometry. The period from 2005 to 2015 marked a surge in CD38 research, with keywords such as breast cancer, colorectal cancer, prostate cancer, apoptosis, hematopoietic stem cells, therapy and gene expression gaining attention. Since 2015, studies have increasingly explored areas, such as immunotherapy, antibody, dexametrasome, pointing toward key directions for future research in the field of CD38-related diseases.

### 3.2 Key insights focused on three investigational axes

#### 3.2.1 Specific age-related disease clusters

**Figure 5:**
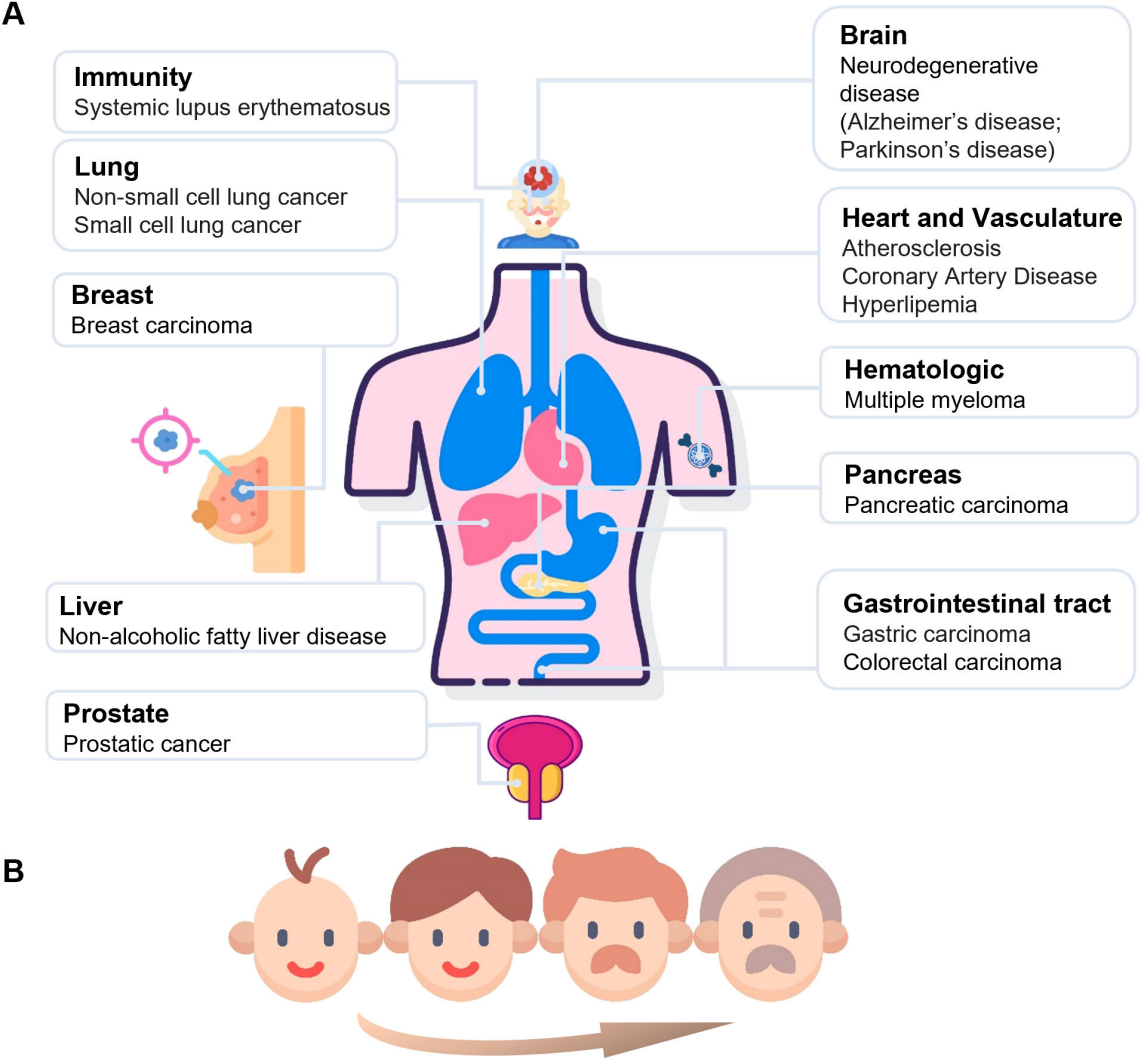
The systemic outcomes of increased CD38 activity across various organs. This figure illustrates the various diseases associated with increased circulating CD38 activity among different organs. A wide range of organs, including the brain, lungs, heart and vascular system, mammary glands, gastrointestinal tract, pancreas, liver, prostate, immune system, and hematologic, are closely related to the increase in CD38 activity in the blood **(A)**. In addition to disease states, enhanced CD38 activity is also associated with aging, with its activity gradually increasing with age **(B)**.

Low NAD levels are significantly associated with aging. Therefore, understanding the effects of NAD metabolism on aging and age-related diseases is important for studying disease mechanisms. Recent studies have shown that CD38 plays a key role in various age-related diseases because of its involvement in NAD metabolism and maintenance of NAD homeostasis (4, 29). In recent years, great interest has been shown in the study of CD38 in aging and age-related diseases, including cancer (5, 30), metabolic diseases (22), and neurodegenerative diseases (29) (**Figure 5A**). Particularly, the potential of CD38 in the treatment of diseases has received much attention.

##### 3.2.1.1 CD38 and aging

Several studies have demonstrated that CD38 plays a crucial role in aging and age-related diseases. The enzymatic activity of CD38 has been shown to positively correlate with age. In a study analyzing serum samples, CD38 activity was found to be normally distributed across different age groups and did not differ significantly between males and females; however, a significant positive correlation between CD38 activity and chronological age was observed (27) (**Figure 5B**). In terms of protein expression, CD38 levels have been shown to increase with aging and are strongly associated with the decline of NAD+ levels. Camacho-Pereira et al. conducted experiments in aged mice and demonstrated that while the activities of other NAD-consuming enzymes such as PARP and SIRT1 remained relatively stable with age, CD38 expression markedly increased (28). Furthermore, they showed that knockdown or pharmacological inhibition of CD38 preserved NAD+ levels and mitochondrial function in aged mice, suggesting a causal role for CD38 in driving age-associated NAD+ decline and metabolic dysfunction (14).

While early hypotheses suggested that NAD+ decline during aging was primarily due to reduced synthesis by enzymes such as NAMPT and NAPRT (31, 32), later studies, including those by McReynolds MR et al., provided evidence that increased NAD+ consumption, rather than impaired synthesis, is a major contributor (33). Supporting this, CD38 expression was observed to increase up to 2.5-fold in the adipose tissue of older human subjects (28), indicating that findings from animal models may extend to humans.

Despite these important insights, the precise molecular mechanisms by which CD38 contributes to aging processes and the development of age-related diseases remain incompletely understood. Questions remain regarding how CD38 is regulated during aging, whether its effects are tissue-specific, and how CD38-targeted interventions might influence the progression of aging-related pathologies.

##### 3.2.1.2 CD38 and cancer

Emerging evidence highlights the association between CD38’s biological features and cancer progression, yet its mechanistic implications remain unresolved. Several studies have demonstrated that CD38 is associated with various types of cancer, including gastric, lung, and breast cancer. Elevated CD38 expression in these cancers has been reported, but the biological significance of this finding remains unclear (26). In particular, CD38 expression in erythrocytes of patients with colorectal, gastric, pancreatic, and breast cancer was found to be significantly higher, with strong correlations to clinical factors such as CEA values, tumor stage, disease progression, lesion site, and degree of anemia. These observations suggest that CD38 may play a role in the inflammatory response to tumors, potentially making changes in CD38 expression relevant for prognostic studies (34, 35).

In contrast, the enzymatic activity of CD38 in serum samples of patients with various cancers was found to be two to three times higher than that in healthy controls, although the exact biological implications of this elevated enzymatic activity and the presence of anti-CD38 proteins in cancer remain unclear (26). Furthermore, studies by Mottahedeh J and Chini CC et al. observed lower CD38 expression and higher NAD+ levels in pancreatic and prostate cancer cells, indicating a paradoxical relationship (36, 37). Despite the reduced expression, CD38 activity increased, and NAD+ levels decreased, leading to diminished tumor cell growth, increased apoptosis, and senescence. These contradictory findings highlight the complex role of CD38 in cancer metabolism and immunomodulation, though its precise function in the tumor microenvironment remains unclear (36–38).

##### 3.2.1.3 CD38 and metabolic diseases

NAD is located at the core of metabolism and its homeostasis is necessary to maintain the normal function of various tissues such as adipose, muscle, gastrointestinal, renal, and hepatic tissues (16). Altered metabolic states such as high-fat diets can decrease NAD levels, which in turn decreases other NAD+-dependent cellular processes (16). CD38 knockout mice or CD38 inhibitor-treated mice exhibited increased metabolic rates and lower risk of metabolic syndromes, such as obesity. They had a normal metabolic state despite aging and receiving high-fat diets (28, 39). It has been shown that decreased NAD^+^ level is associated with senescence-associated secretory phenotype (SASP). Furthermore, it has been experimentally demonstrated that a SASP-conditioned medium induces CD38 expression in macrophages and endothelial cells (40). Chini C et al. found that senescent cells and SASP activate CD38 expression in macrophages and enhance the function of CD38 (41). Although increased CD38 expression in metabolically abnormal cells has been well documented, further studies are needed to investigate the exact role of CD38 in metabolic diseases.

##### 3.2.1.4 CD38 as a potential target for treatment

With the in-depth study of the role of CD38 in various diseases, increasing attention has been given to targeting CD38 as a therapeutic strategy. Different pharmacological approaches have been developed, including cytotoxic antibodies, enzyme-blocking antibodies, and small molecule inhibitors (14, 42). Recent studies have further established that CD38 monoclonal antibodies, such as daratumumab and isatuximab, can induce apoptosis of myeloma cells, promote clonal T-cell expansion, and modulate the tumor immune microenvironment, thus supporting their role as effective immunotherapeutic agents (38, 43). Dalertuximab, a humanized monoclonal antibody targeting CD38, has demonstrated anti-tumor activity and has been approved for the treatment of multiple myeloma by mediating antibody-dependent cellular cytotoxicity (ADCC) and modulating the tumor immune microenvironment (14, 44). It binds to CD38 on MM cells, exerts its ectoenzyme hydrolytic activity to generate metabolites that regulate intracellular calcium levels and activate purinergic receptors, thereby leading to cell lysis and immune-mediated tumor suppression. This strengthens the rationale for targeting CD38 in the treatment of MM and potentially other CD38-expressing malignancies (45). Additionally, some covalent CD38 inhibitors have shown enhanced tumor-suppressive effects in isolated Th1/17 hybrid cells, highlighting their potential as CD38-targeted modulators in cancer therapy (14).

#### 3.2.2 The mechanisms by which CD38 affects the aging process via NAD metabolism

**Figure 6:**
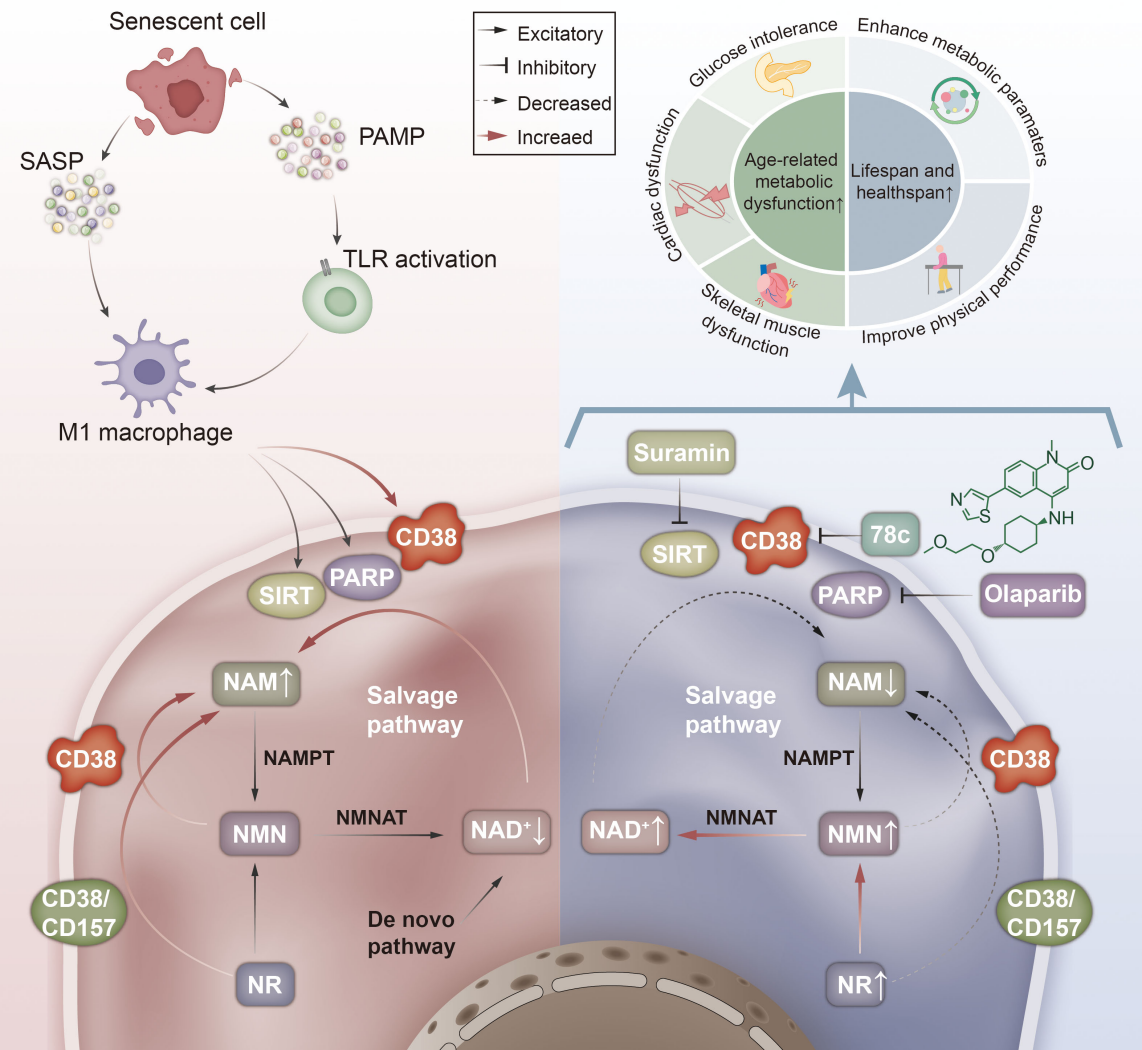
Mechanisms of CD38 activity changes and aging. This figure illustrates the biological mechanisms by which changes in CD38 activity impact aging processes. Secretion of SASP by senescent cells or activation of innate immune cells via PAMP promotes macrophage M1 polarization thereby significantly enhancing CD38 activity in M1 macrophages and increasing hydrolysis of NAD to generate NAM, while the NAD precursors NMN and NR also generate NAM **(A)**; while inhibition of using the CD38-specific inhibitor 78c significantly reduces this process, reducing NAD hydrolysis and thereby enhancing age-related metabolic dysfunction and prolonging the lifespan and health span**(B)**. SASP, Senescence-associated secretory phenotype; PAMP, Pathogen-associated molecular patterns; TLR, Toll-like receptors; SIRT, Sirtuin; PARP, poly ADP-ribose polymerase; NAD, Nicotinamide adenine dinucleotide; NAM, Nicotinamide; NMN, Nicotinamide mononucleotide; NR, Nicotinamide riboside; NMNAT, Nicotinamide mononucleotide adenylyl transferase; NAMPT, Nicotinamide phosphoribosyl transferase.

Although there has been a strong interest in NAD metabolism in aging, and it was shown that the accumulation of CD38(+) cells plays an important role in aging-associated decrease in NAD+ levels (32), studies so far have indicated that CD38 influences NAD+ depletion through multiple pathways, including its activity in senescent cells and macrophages, which affects both the salvage and *de novo* NAD+ biosynthesis pathways (**Figure 6**). However, the specific mechanisms by which CD38 affects NAD levels and the aging process needs further studies.

##### 3.2.2.1 Mechanisms associated with the involvement of CD38 in NAD metabolism

Covarrubias et al. investigated how acute and chronic inflammation decreases macrophage CD38 expression and NAD levels in metabolic tissues. Cellular stressors, such as DNA damage, lead to the accumulation of senescent cells over time and induce the release of essential SASP. Increased intestinal permeability during senescence increases the serum levels of endotoxin and other PAMPs, which activate innate immune cells. SASP and PAMP promote macrophage M1-like polarization, thereby increasing CD38 expression in macrophages up to 600-fold in tissues, whereas SIRT1, PARP1, and PARP2 are upregulated to a lower extent (46).

NAD can be synthesized *de novo* from tryptophan or through the NAM salvage pathway. Measurement of the mRNA levels of the key enzyme for *de novo* pathway synthesis, indoleamine 2,3-dioxygenase1, and the key enzymes for salvage pathway synthesis, NAMPT, and NMNAT, indicated a significant increase in NAMPT and NMNAT1. It maintains the level of nuclear NAD and regulates the level of NAD in the cytoplasm and M1 macrophages. The levels of NMNAT2 and NMNAT3, which regulate NAD levels in mitochondria, did not significantly change. In contrast, kynurenine 3-monooxygenase and 3-hydroxyanthranilic acid oxygenase, two key enzymes for *de novo* synthesis, were markedly downregulated (16, 46). These results suggest that the NAD salvage pathway is an important regulator of macrophage polarization and fine-tuned gene expression. CD38 and CD157 can be used as alternative substrates in their catalytic reactions, degrading NMN to generate NAM and ribose monophosphate (RMP), and degrading NR to generate NAM and ribose (16) (**Figure 6**).

In contrast, the CD38 enzyme-specific inhibitor 78c significantly increased NAD levels without significantly changing the expression of genes involved in NAD+ catabolism and anabolism, suggesting that 78c increases NAD+ levels through the catalytic activity of the CD38-dependent NAD enzyme, rather than through the action of other NAD+-metabolizing enzymes. Similarly, NAD+ levels increased when using suramin, an inhibitor of SIRT activity, or olaparib, a PARP inhibitor. Their combination even showed a greater impact (39). Several studies have shown that the specific inhibitor 78c possesses significant health-promoting effects in aging, ameliorates age-related metabolic dysfunction, and increases lifespan and health span (39, 47). By measuring NAD levels in mice treated with 78c, Mariana et al. noted an improvement in glucose homeostasis in older mice but not in younger mice. Similarly, both age-associated skeletal muscle dysfunction and age-associated cardiac dysfunction significantly improved after treatment with 78c (39) (**Figure 6**). Thais R. Peclat et al. conducted an *in vitro* longitudinal experiment and indicated that treatment with 78c can promote motor performance and enhance metabolic function, suggesting the potential of CD38 inhibitor 78c for age-related diseases (47).

##### 3.2.2.2 Mechanisms related to the role of CD38 in the aging

Based on the twelve hallmarks of senescence identified in the journal CELL (48), in addition to cellular senescence and chronic inflammation, CD38 may play an important role in genomic instability, mitochondrial dysfunction, and macroscopic autophagy dysfunction, thereby promoting mammalian senescence (49).

NAD-dependent silent information regulator acetylase (SIRT) appears to be an important regulator of genomic stability (30), and there are no reports on the direct role of CD38 in this senescence trait. For the first time, Camacho-Pereira J et al. identified a significant increase in the mitochondrial levels of the silencing regulator protein SIRT3 in the liver of 1-year-old wild-type and CD38 knockout mice. Although comparable, the mitochondrial levels of the silencing regulator protein SIRT3 were significantly higher in wild-type mice than in CD38 knockout mice (28). In addition, in the pre-senescent of endogenous NAD, the SIRT3 activity of CD38 knockout mice was 3.5-fold higher than that of wild-type mice. When the saturating levels of NAD were added for enzymatic assay, there was no significant difference between the SIRT3 activities of wild-type mice and knockout mice (41, 50). Together, these data suggest that CD38 may alter cellular NAD through regulation-dependent SIRT3 activity rather than SIRT3 levels (28). Wang LF et al. reported that SIRT1 inhibition can partly reverse the effect of CD38 knockout on D-galactose-induced cardiomyocyte senescence, suggesting that CD38 attenuates senescence and oxidative stress through the NAD/SIRT1 signaling pathway (51). SIRT1 is the most prominent member of the deacetylase family, and the enhanced anti-tumor properties of Th1/17 cells rely on the enhanced activity of the NAD-dependent enzyme SIRT1. SIRT1 is closely associated with the differentiation of Th1/17 cells. Reduced activity of CD38 increases the bioavailability of NAD and activates SIRT1, thereby regulating the growth and differentiation of T cells and tumor growth. These results suggest that the CD38-NAD-SIRT1 axis may be a target for treating cancer and infection (16, 52, 53).

At the same time, declined mitochondrial function is a hallmark of aging, and decreased cellular NAD+ levels have been suggested as a potential cause of this age-related mitochondrial dysfunction. Significant increases in mitochondrial oxygen consumption and activity were found in the liver tissue and spleen mitochondria from CD38-deficient mice, with no significant differences in their numbers. However, the mRNA expression levels of Pdha1, PdK2, glycolytic/pentose pathway enzymes, and small solute carriers decreased, suggesting that the increase in oxygen consumption is associated with an increase in mitochondrial NAD rather than an increase in mitochondrial biosynthesis. CD38 overexpression in cells reduced the total respiratory capacity, increased dependence on glycolysis and abnormal mitochondrial morphology, which may be due to CD38 degradation and/or the leakage of NAD+ and NADH from dysfunctional mitochondria during *in situ* or mitochondrial detachment (54). Mitochondria, as the most sensitive organelles, were shown to be structurally and functionally impaired in acute myocardial ischemia, characterized by decreased NAD+. Besides, inhibition of the CD38 enzyme by NAM reduced NAD+ degradation (55, 56). *In vivo* experiments have also demonstrated that the administration of exogenous NAD+ to CD38-deficient mice protects the heart against ischemia/reperfusion and reduces the size of the infarct (57). These results suggest that CD38 may regulate NAD+ levels in coronary artery disease. The role of CD38 in the pathogenesis of atherosclerosis via NAD metabolism remains controversial. Xu et al. found that after receiving 12 weeks of Western diet, CD38-deficient mice exhibited more severe atherogenesis compared to wild-type mice (58). In a follow-up study, it was found that increased autophagy in CD38-deficient myocytes exacerbated coronary fibrosis and atherosclerosis in mice (59). However, Becerio et al. found that transplantation of CD38-deficient bone marrow into Ldlr^-/-^ mice can prevent the development of aortic atherosclerosis (59). In addition, Kong XY et al. confirmed that CD38 deficiency did not alter the risk of atherosclerosis (21). These results suggest that regulation of NAD homeostasis through changes in CD38 expression can finally affect the function of mitochondria, and that mitochondrial dysfunction is crucial in aging.

It has also been reported that CD38 is involved in autophagy dysfunction. Mutations in the LRP2 gene are risk factors for Parkinson’s disease, Crohn’s disease, leprosy, and some cancers whose pathogenesis is associated with macro-autophagy/autophagy. Neel R Nabar et al. linked CD38 and LRPK2 as upstream activators of the transcription factor EB in immune cells. They found that the physiological CD38-LRPK2-TFEB signaling axis that functions by activating the downstream lysosomal transcriptional regulator TFEB is involved in autophagy (60).

#### 3.2.3 The clinical application of CD38

To deeply study the function of CD38 and its role in diseases, various detection methods have been developed to measure the expression and activity of CD38, such as molecular biology, biochemical analysis, and imaging. Among them, techniques such as flow cytometry, Western blotting, immunohistochemistry, and high-performance liquid chromatography have been widely applied in the detection of CD38 (**Table 1**).

**Table 1:** Summary of CD38 detection methods.

Analytical Parameters	Detection method	Principle	Sample	Features	Limitations
Protein Level	Western Blotting	Protein separation and detection; Specific antigen-antibody binding	Cells (28, 34, 51) Tissues	Broad application; Simple operation	Time-consuming; Limited sensitivity; Technical variability; Subjectivity in interpretation; Relative quantitation; Cross-Reactivity Issues
	Immunohistochemical	Specific antigen-antibody binding; Labeled antibody staining;	Cells Tissues (61, 62)	Visualization; Simultaneous localization and quantification	
	Flow Cytometry	Laser illumination; Scattered light and fluorescence signal detection; Multiparameter cell analysis	Cells Tissues Blood (63)	High-speed analysis; High sensitivity; Cell sorting capability	
	Fluorescence Microscope	Antibody fluorescence labeling; Specific antigen-antibody binding; Fluorescence signal observation	Cells Tissue (63)	High sensitivity; Multichannel detection; Real-time dynamic observation and localization	
Enzyme Activity	Enzyme Cycling Assay	Enzymatic catalysis specificity; Product regeneration cycle; Signal amplification; Indirect quantitative analysis	Cells (36)	Signal Amplification; Broad Dynamic Detection Range; Higher sensitivity; Absolute quantitation	Limited applicability; Time-consuming; Susceptible to interference
	Thin-Layer Chromatography	Sample separation; Solvent migration; Component detection	Serum Erythrocyte (34)	Simple and rapid; Strong separation ability; High sensitivity; Absolute quantitation	Limited resolution; Time-consuming; Susceptible to interference
	Fluorescence Photometry	Enzymatic catalysis specificity; Fluorescence signal change	Cell (36)	Wide dynamic range; Real-time dynamic observation; Highest sensitivity; Absolute quantitation	High background signal; Susceptible to environmental factors; Fluorescence quenching phenomenon
	High-Performance Liquid Chromatography	Substrate-specific conversion; Quantitative separation of products	Erythrocyte (27, 64) Plasma (65)	High resolution; High-speed analysis; Ultimate sensitivity; Absolute quantitation	High cost; High technical requirements

##### 3.2.3.1 Protein level measurement methods

Detection of CD38 expression by flow cytometry is affected by the sensitivity of the fluorescent dye binding reagent. A study reported the frequency and average fluorescence intensity of CD38 expression when using phaeoglobin and fluorescein isothiocyanate-labelled anti-CD38 monoclonal antibodies alone or in combination with human leukocyte antigen DR. The study aimed to determine the expression of CD38 by the two stains in different human immunodeficiency virus-infected patients at different levels (63). The results showed that although both stains detected high frequencies of CD38-expressing cells, only the phaeoglobin-conjugated reagent correlated with markers of disease progression. In contrast, fluorescein isothiocyanate-conjugated reagent could not help monitor the increase in CD38 expression in patients. Therefore, careful selection of fluorescent dye binding reagents is essential for quantifying CD38 in clinical settings. Western blotting has become a common tool for protein detection due to its simplicity and low cost. Several studies have confirmed that CD38 is a protein band with a size of 45 kDA (28, 51). Immunohistochemical methods can visualize the location and expression level of CD38 in tissues or cells, thus providing detailed information about its distribution and expression during physiological and pathological processes (61, 62). However, this method can only detect the protein expression level of CD38 and does not accurately determine the activity of CD38 as an enzyme. Although compared to quantitative methods such as CD38 content determination, the enzyme activity of CD38 can better reflect its biological function.

##### 3.2.3.2 Enzyme activity assay

Current methods for determining the enzymatic activity of CD38 include enzyme cycling, thin-layer chromatography, and fluorescence photometry. However, fluorescence photometric detection of enzyme activity necessitates the substrate containing radioactive isotopes to undergo an enzymatic reaction with the sample. The amount of eluted radioactive isotopes in the product is counted using liquid scintillation to calculate the enzyme activity (36). These enzyme activity assay methods are not only cumbersome in operation but also have low determination accuracy. High-performance liquid chromatography (HPLC) has been widely used due to its high resolution, high sensitivity, and fast analysis. Polzonetti et al. used a reversed-phase C18 column to conduct gradient elution of the mixture after the enzymatic reaction. They monitored the absorbance of the product at 254 nm, and finally calculated CD38 by determining the amount of the product and residual substrate based on the peak area of the compounds separated using HPLC enzyme activity (27). This method still has more shortcomings. Firstly, the need to terminate the reaction at different time points increases the complexity of the assay. Secondly, the longer gradient elution prolongs the assay. The method introduced by Kirchberger T et al. for determining the enzymatic activity of CD38 by HPLC also showed that reversed-phase C18 columns are suitable for separating the product. However, the method only describes the pretreatment method for the enzymatic activity of the surface of tissues or cells. They are not fully applicable to blood samples, which are more commonly used in clinical practice (64, 65). Overall, high-performance liquid chromatography (HPLC) plays a key role in studying the enzymatic activity of CD38. Continuous method optimization and technological improvements have improved the accuracy and sensitivity of the assay, providing a powerful tool for in-depth assessment of the biological function of CD38 in disease pathogenesis. Accurate and sensitive HPLC enzymatic assays are needed to obtain a more comprehensive insight into the enzymatic activity of CD38, achieve high-throughput, simple, accurate, and reliable detection results, and serve epidemiological studies and clinical applications.

## 4 Discussion

This investigation implemented an integrative scientometric-analytical framework to delineate evolving research paradigms and conceptual frontiers in CD38-related aging pathobiology. The findings indicated a steady and exponential increase in the number of publications over years, underscoring the critical role of CD38 in aging research. Co-occurrence and clustering analyses of keywords revealed that current research is predominantly focused on hematological diseases and cancer, providing invaluable insights into potential future research directions. To our knowledge, this is the first original investigation to combine bibliometric analysis with a detailed examination of the role of CD38 in aging and age-related diseases, offering significant visualization and clarification of research priorities and directions.

Our study provides a valuable resource by compiling and analyzing a comprehensive dataset of research articles. This dataset offers a reference for researchers interested in the biological and pathological roles of CD38. By elucidating the existing research landscape, this work serves as a guide for future studies aiming to deepen our understanding of the involvement of CD38 in age-related processes.

Our analysis revealed a marked increase in the number of publications on CD38 in aging and age-related diseases from 2009, with a subsequent exponential growth pattern. This trend reflects an evolving understanding of the biological significance of CD38 and a growing need to explore its role in aging processes. Notably, this surge in research activity is consistent with the global increase in aging populations (66), emphasizing the increasing need to address age-related health challenges. The geographical distribution of publications indicates that the majority of studies originated from economically advanced countries, including the United States, China, Italy, Germany, and the United Kingdom. In contrast, contributions from low-income and middle-income countries remain limited, suggesting a potential disparity in research capacity and resources. This underscores the need for broader, more inclusive research efforts that incorporate diverse populations and global perspectives, particularly given the universal effect of age-related diseases (67). Regarding the distribution of journals, a significant proportion of articles have been published in hematology and oncology journals (68, 69). This indicates that the role of CD38 in hematological malignancies and cancer has been extensively studied, likely due to its established involvement in conditions such as leukemia, lymphoma, and multiple myeloma. However, despite the dominance of these fields, the presence of age-related keywords in bibliometric analyses suggests that CD38 is involved in diverse fields other than hematology and oncology, warranting further studies into its broader implications in aging biology.

Although this study focused on the role of CD38 in aging and related diseases, the keyword co-occurrence and clustering analysis highlighted hematological disorders as the dominant research area. The absence of aging-specific clusters may reflect the current research emphasis on the role of CD38 in blood-related diseases, potentially biasing the results. This could be due to the larger volume of studies exploring the role of CD38 in hematological diseases compared to age-related diseases. Nevertheless, keywords such as aging, cellular senescence, and oxidative stress emerged in the co-occurrence analysis, suggesting that the role of CD38 in aging remains a relevant and emerging area of interest (70, 71). These findings suggest that although the involvement of CD38 in hematological diseases has been more extensively studied, its role in aging is gaining recognition and offers promising avenues for future research.

Importantly, recent studies have further expanded the scope of CD38 research into emerging fields such as stem cell biology and infectious diseases (72, 73). In stem cell research, CD38 has been shown to modulate hematopoietic stem cell aging and exhaustion, implicating it in regenerative decline and age-associated dysfunction of the hematopoietic system (73). In parallel, the COVID-19 pandemic has highlighted CD38’s immunological relevance, particularly as a marker of immune activation in severe cases (15, 72). Elevated CD38 expression on immune cells during viral infection points to its role in immune dysregulation, inflammation, and possibly immune senescence (74). These findings underscore a dynamic shift in the field, where CD38 is increasingly studied not only in chronic degenerative diseases but also in acute systemic pathologies. As such, future studies should consider these novel directions to more comprehensively capture the evolving and multifaceted role of CD38 in health and disease.

In summary, this study provides a comprehensive bibliometric and literature-based assessment of CD38 in the context of aging. Despite the current dominance of hematological research, the presence of age-related and newly emerging keywords underscores the expanding relevance of CD38 in multiple biological contexts. These findings highlight the potential for deeper exploration of CD38’s role in aging and related pathologies, and suggest promising directions for future investigation across a broader range of disease models and biological systems.

## 5 Conclusion and limitations

This article employs a dual-modality analytical framework to systematically decode the pathophysiological of CD 38 in aging-associated pathologies. As a major NAD-depleting enzyme, CD38 plays a crucial role in cellular function and disease pathogenesis. While the biological role of CD38 in disease pathogenesis has garnered significant attention, along with the development of several assay methods, current studies have some limitations. Although CD38 is expressed in various cells, such as immune cells, erythrocytes, and endothelial cells, there are limited reports on its activity differences among these cells. Future studies on the differences in its activity in different cells may provide important references to the role of CD38 in metabolism. In addition, the structural domain of CD38 is located in the extracellular cell, but its main biological functions occur in the intracellular space, regulating the level of intracellular NAD, also known as the ‘topological paradox’ of CD38. The reason for this phenomenon and the mechanism by which it regulates cellular metabolism have not been elaborated in detail. Further studies on CD38 detection tools and mechanisms can help reveal the deeper relationship between CD38 and diseases and develop relevant therapeutic strategies.

However, this study had certain limitations. Firstly, the analysis was based solely on publications indexed in the Web of Science database. Although comprehensive, this may exclude relevant studies from other databases or non-English sources, introducing potential selection bias. Secondly, although the primary focus of this study was to investigate the relationship between CD38 and aging or age-related diseases, the broad scope of age-related diseases presents a challenge. These diseases encompass a wide range of conditions, and our analysis provides a high-level overview rather than an in-depth examination of the role of CD38 in each specific disease. Future studies should consider more detailed investigations into the relationship between CD38 and individual diseases within the aging spectrum to provide a more nuanced understanding. Despite these limitations, this study offers valuable insights into the current research landscape of CD38 in aging, highlighting both established research areas and emerging trends.

## Data Availability

The original contributions presented in the study are included in the article/**Supplementary Material**. Further inquiries can be directed to the corresponding authors.
