# Supplementary material for: Unveiling the role of NAD glycohydrolase CD38 in aging and age-related diseases: insights from bibliometric analysis and comprehensive review
Source: Front Immunol. 2025 Jun 2;16:1579924. doi: 10.3389/fimmu.2025.1579924 (PMC12171214; doi:10.3389/fimmu.2025.1579924)
Supplement: Supplementary file 1 [file DataSheet1.docx]

**Knowledge mapping and research trend of NAD Glycohydrolase CD38 in Aging and Age-Related Diseases from 2004 to 2023: a bibliometric analysis**

Supplement Materials


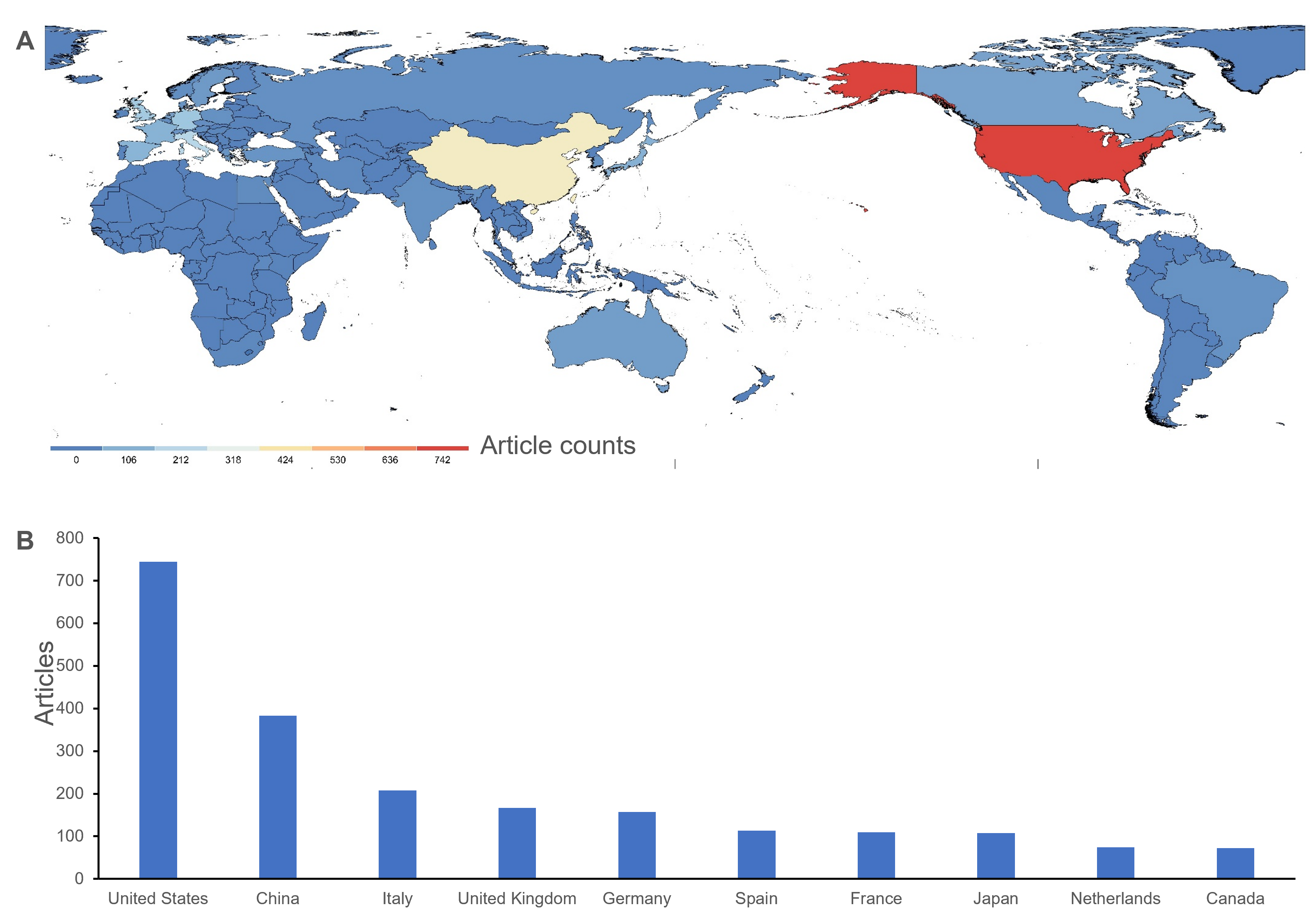


**Fig.S1.** Each country’s Contribution to the CD38 in Aging and Age-related Diseases.

A. Heat map of country distribution for published articles; B. Top 10 countries by publication count.


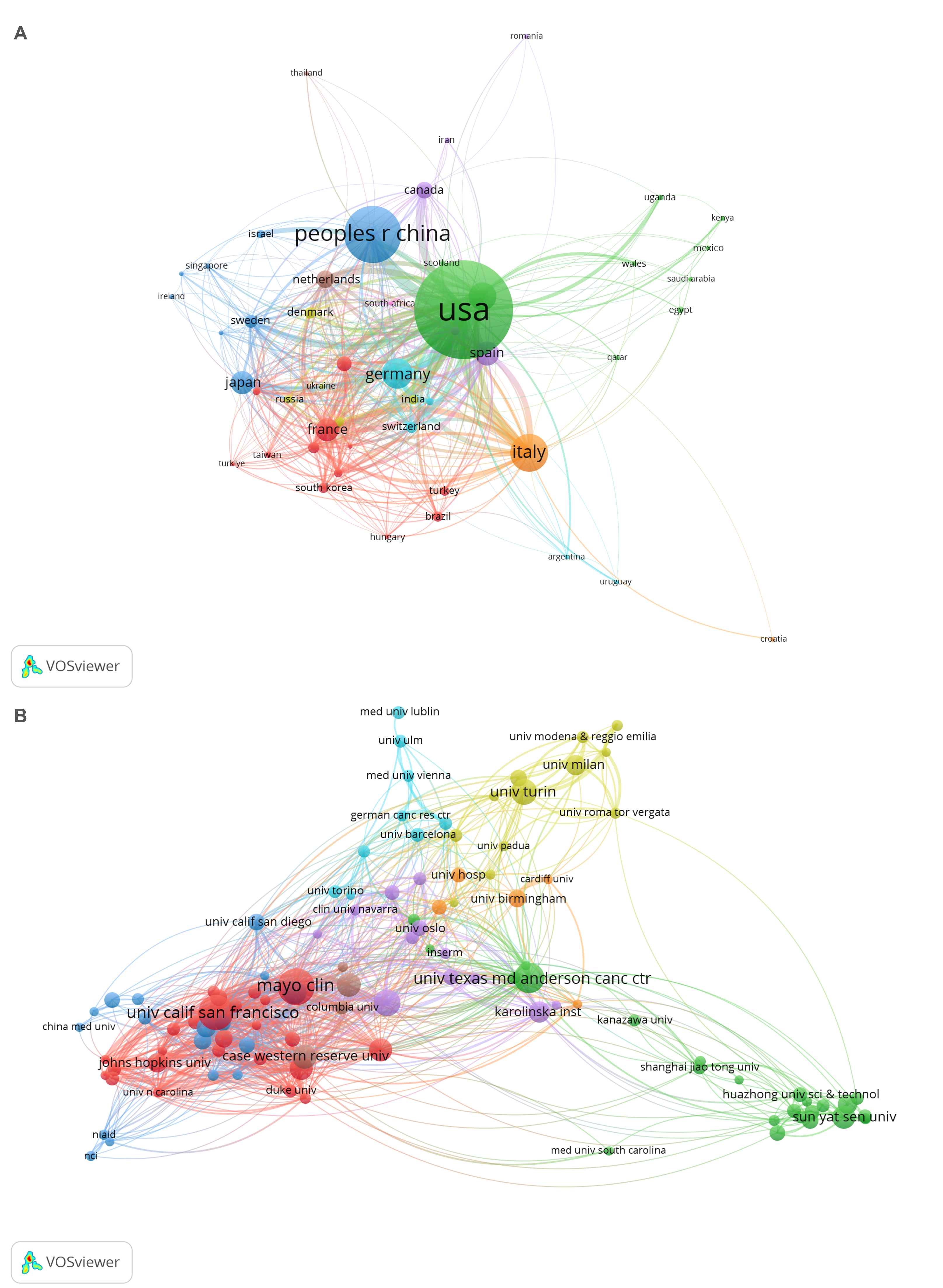


**Fig.S2.** The Distribution of Countries and Institutions Publishing Research on CD38 in Aging and Age-related Diseases.

A. Distribution by countries/regions. B. Distribution by institutions.


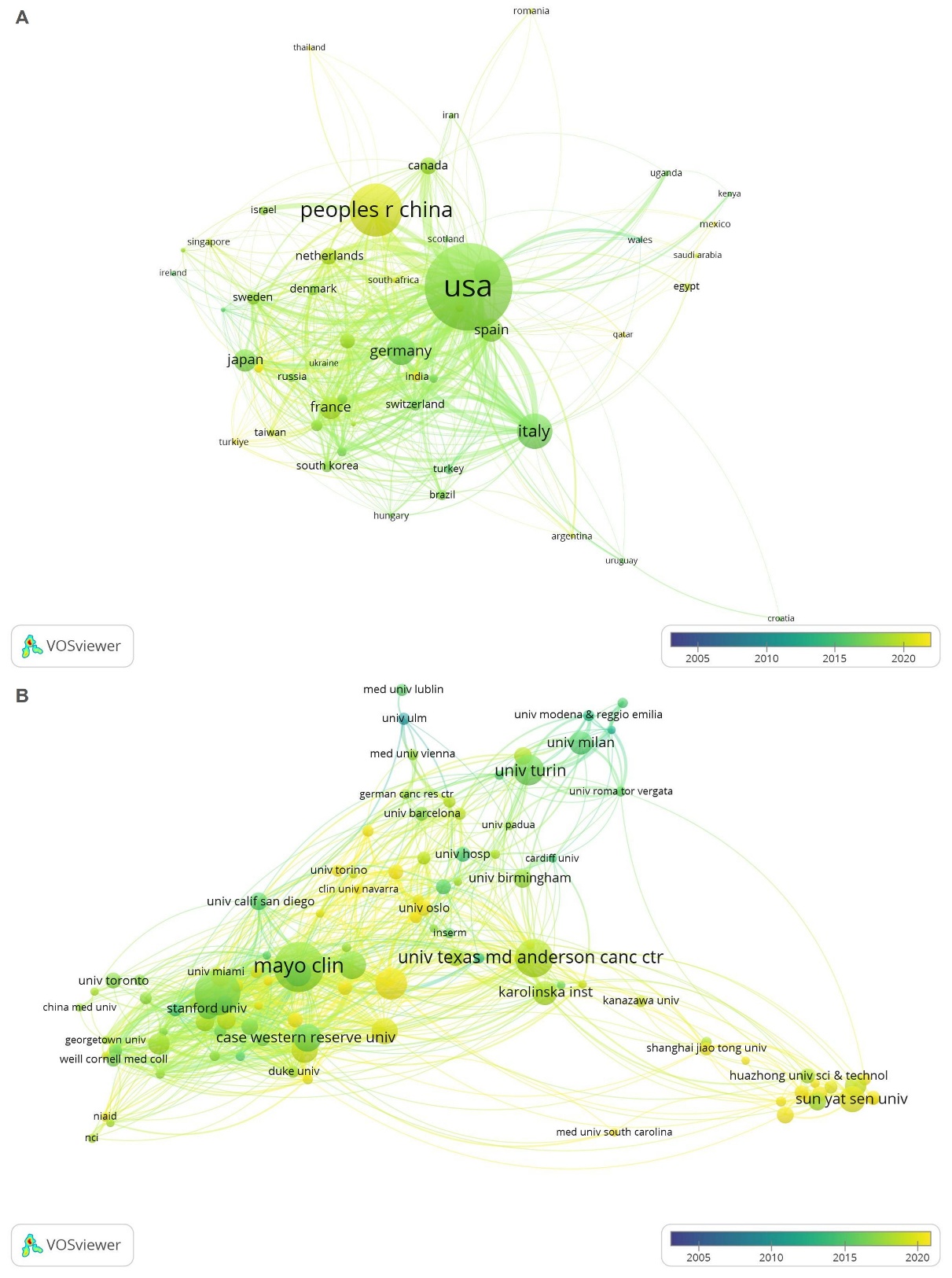


**Fig. S3.** The Time-overlapping Map of Countries/Regions and Institutions Publishing Research on CD38 in Aging and Age-related Diseases.

A. Distribution by countries/regions. B. Distribution by institutions.


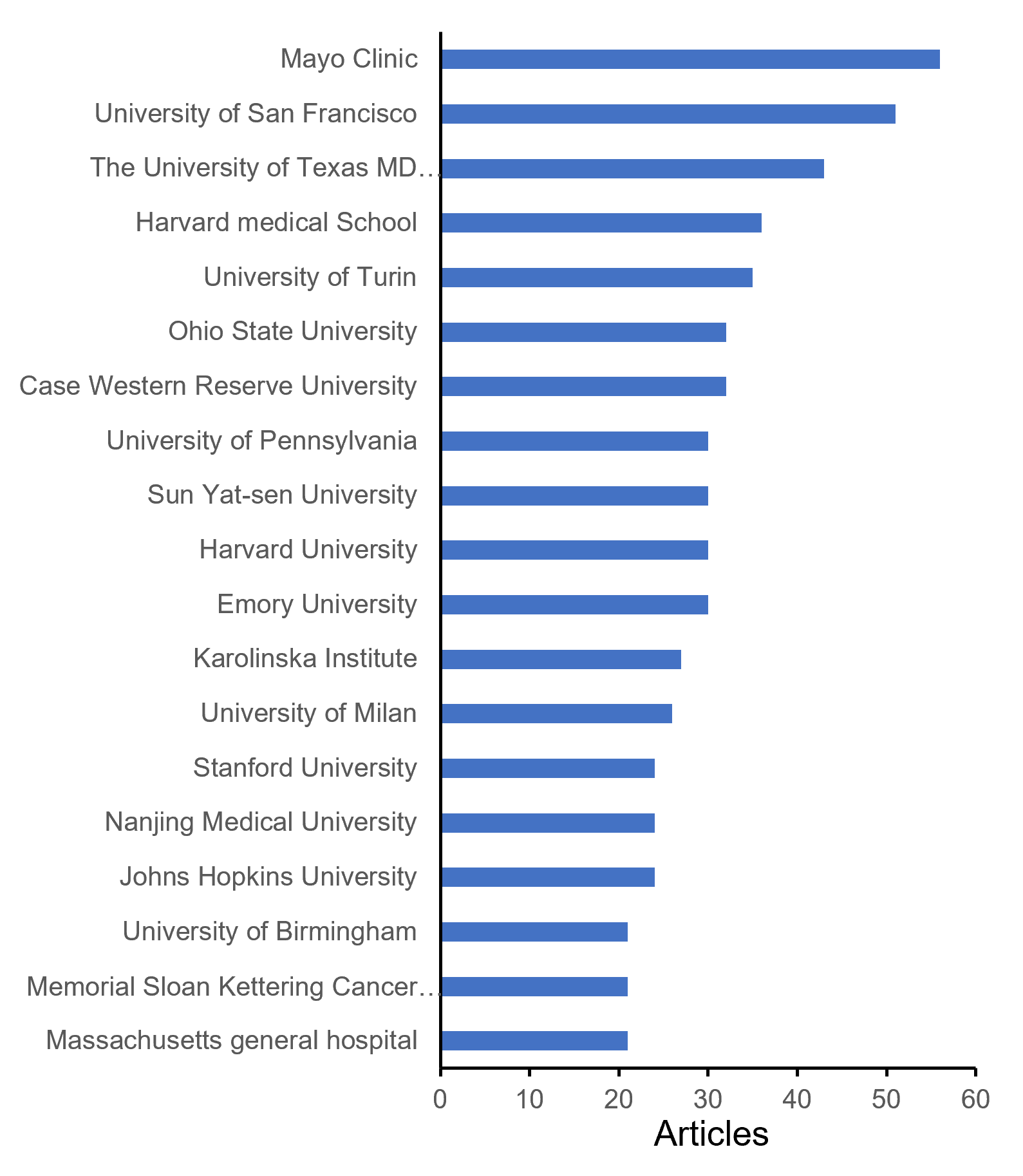


**Fig.S4.** The Top 19 Institutions with the Most Articles in the Fields of CD38 in Aging and Age-related Diseases.


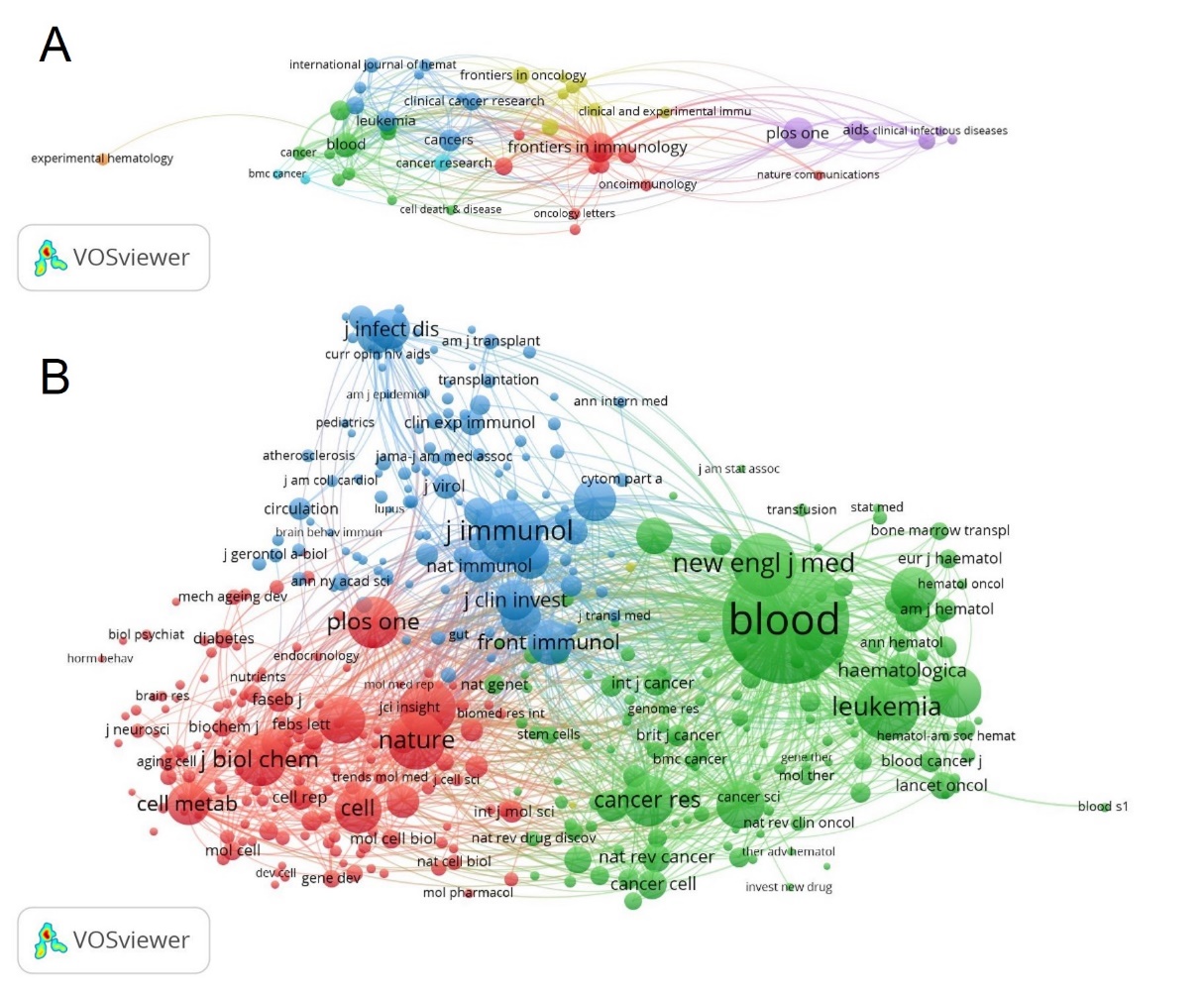


**Fig. S5.** Journal and Co-citation Network Publishing Research on CD38 in Aging and Age-related Diseases.

A. Article-publishing journals; B. Journals citing related articles.

**Table S1**

Top 10 Journals for Research of CD38 in Aging and Age-related Diseases.

| Rank | Source | Article | Country | IF | JCR-c |
| --- | --- | --- | --- | --- | --- |
| 1 | Frontiers in Immunology | 69 | Switzerland | 5.7 | Q1 |
| 2 | Plos One | 67 | USA | 2.9 | Q1 |
| 3 | Blood | 42 | USA | 21 | Q1 |
| 4 | Cancers | 39 | Switzerland | 4.5 | Q1 |
| 5 | Aids | 30 | USA | 3.4 | Q2 |
| 6 | Leukemia | 30 | UK | 12.8 | Q1 |
| 7 | Leukemia & Lymphoma | 28 | UK | 2.2 | Q3 |
| 8 | Scientific Reports | 26 | England | 3.8 | Q1 |
| 9 | Frontiers in Oncology | 24 | Switzerland | 3.5 | Q2 |
| 10 | International Journal of Molecular Sciences | 24 | Switzerland | 4.9 | Q1 |
|  |  |  |  |  |  |

**Table S2**

Top 10 Co-cited Journals for Research of CD38 in Aging and Age-related Diseases.

| Rank | Source | Co-citations | Country | IF | JCR-c |
| --- | --- | --- | --- | --- | --- |
| 1 | Blood | 8515 | USA | 21 | Q1 |
| 2 | Journal of Immunology | 2293 | USA | 5.4 | Q2 |
| 3 | New England Journal of Medicine | 2250 | USA | 96.2 | Q1 |
| 4 | Leukemia | 2198 | UK | 12.8 | Q1 |
| 5 | Nature | 1849 | UK | 50.5 | Q1 |
| 6 | Proc Natl Acad Sci USA | 1694 | USA | 9.4 | Q1 |
| 7 | Journal of Clinical Oncology | 1504 | USA | 42.1 | Q1 |
| 8 | Plos One | 1471 | USA | 2.9 | Q1 |
| 9 | Journal of Biological Chemistry | 1416 | USA | 4.0 | Q2 |
| 10 | Frontiers in Immunology | 1391 | Switzerland | 5.7 | Q1 |

**Table S3**

The Top Authors in the Field of CD38 in Aging and Age-related Diseases Ranked by Publication and Citation Numbers.

| Author | Documents | Co-cited Author | Citations |
| --- | --- | --- | --- |
| malavasi, fabio | 23 | malavasi, f | 255 |
| chini, eduardo n. | 16 | damle, rn | 236 |
| mccomsey, grace a. | 15 | dimopoulos, ma | 230 |
| horenstein, alberto l. | 13 | hamblin, tj | 229 |
| moreau, philippe | 13 | deaglio, s | 224 |
| zent, clive s. | 12 | van de donk, nwcj | 222 |
| chini, claudia c. s. | 11 | moreau, p | 180 |
| lederman, michael m. | 11 | hallek, m | 173 |
| medeiros, l. jeffrey | 11 | döhner, h | 167 |
| morabito, fortunato | 11 | horenstein, al | 159 |
| shanafelt, tait d. | 11 | chini, en | 152 |
| xu, wei | 11 | palumbo, a | 148 |
| anderson, kenneth c. | 10 | lee, hc | 143 |
| call, timothy g. | 10 | lonial, s | 137 |
| deeks, steven g. | 10 | mateos, mv | 134 |
| kay, neil e. | 10 | hunt, pw | 133 |
| landay, alan l. | 10 | rai, kr | 132 |
| lonial, sagar | 10 | cantó, c | 128 |
| van de donk, niels w. c. j. | 10 | camacho-pereira, j | 125 |

**Table S4**

Top 10 Documents in Citation Analysis of Publications on CD38 in Aging and Age-related Diseases.

| Rank | Title | First author | Corresponding author | Source | Publication year | Total Citation |
| --- | --- | --- | --- | --- | --- | --- |
| 1 | Ig V gene mutation status and CD38 expression as novel prognostic indicators in chronic lymphocytic leukemia | Damle, R N | N Chiorazzi | Blood | 1999 | 190 |
| 2 | Evolution and function of the ADP ribosyl cyclase/CD38 gene family in physiology and pathology | Malavasi, Fabio | Aydin, S | Physiological Reviews | 2008 | 135 |
| 3 | Unmutated Ig V(H) genes are associated with a more aggressive form of chronic lymphocytic leukemia | Hamblin, T J | Stevenson, F K | Blood | 1999 | 130 |
| 4 | CD38 Dictates Age-Related NAD Decline and Mitochondrial Dysfunction through an SIRT3-Dependent Mechanism | Camacho-Pereira, J | Chini, EN | CELL METABOLISM | 2016 | 124 |
| 5 | Genomic aberrations and survival in chronic lymphocytic leukemia | Dohner, H | Lichter, P | The New England journal of medicine | 2000 | 117 |
| 6 | ZAP-70 expression as a surrogate for immunoglobulin-variable-region mutations in chronic lymphocytic leukemia | Crespo, Marta | Montserrat, Emili | The New England journal of medicine | 2003 | 107 |
| 7 | Daratumumab, a Novel Therapeutic Human CD38 Monoclonal Antibody, Induces Killing of Multiple Myeloma and Other Hematological Tumors | de Weers, Michel | Parren, Paul W. H. I. | JOURNAL OF IMMUNOLOGY | 2011 | 99 |
| 8 | Targeting CD38 with Daratumumab Monotherapy in Multiple Myeloma | Lokhorst, H. M. | Richardson, P. G. | NEW ENGLAND JOURNAL OF MEDICINE | 2015 | 95 |
| 9 | Clinical staging of chronic lymphocytic leukemia. | Rai, K R | Pasternack, B S | Blood | 1975 | 92 |
| 10 | National Cancer Institute-sponsored Working Group guidelines for chronic lymphocytic leukemia: revised guidelines for diagnosis and treatment | Cheson, B D | Rai, K R | Blood | 1996 | 90 |

**Table S5**

Keywords of Documents on CD38 in Aging and Age-related Diseases.

| Rank | Keyword | Occurrences |
| --- | --- | --- |
| 1 | cd38 | 395 |
| 2 | expression | 383 |
| 3 | cancer | 337 |
| 4 | cd38 expression | 210 |
| 5 | survival | 193 |
| 6 | activation | 173 |
| 7 | multiple myeloma | 156 |
| 8 | disease | 139 |
| 9 | t-cells | 137 |
| 10 | cells | 131 |
| 11 | inflammation | 129 |
| 12 | therapy | 126 |
| 13 | immunotherapy | 123 |
| 14 | daratumumab | 105 |
| 15 | prognosis | 105 |
| 16 | chronic lymphocytic leukemia | 103 |
| 17 | immune activation | 103 |
| 18 | lymphocytes | 91 |
| 19 | diagnosis | 87 |
| 20 | acute myeloid-leukemia | 83 |
| 21 | apoptosis | 83 |
| 22 | hiv | 83 |
| 23 | risk | 80 |
| 24 | chronic lymphocytic-leukemia | 79 |
| 25 | cll | 78 |
| 26 | dexamethasone | 78 |
| 27 | multiple-myeloma | 78 |
| 28 | differentiation | 75 |
| 29 | b-cells | 72 |
| 30 | responses | 72 |
| 31 | progression | 68 |
| 32 | gene | 67 |
| 33 | stem-cells | 67 |
| 34 | identification | 66 |
| 35 | open-label | 66 |
| 36 | bortezomib | 65 |
| 37 | flow cytometry | 64 |
| 38 | metabolism | 62 |
| 39 | antiretroviral therapy | 61 |
| 40 | genomic aberrations | 60 |
| 41 | bone-marrow | 58 |
| 42 | gene-expression | 58 |
| 43 | zap-70 expression | 58 |
| 44 | receptor | 57 |
| 45 | aging | 56 |
| 46 | infection | 56 |
| 47 | lenalidomide | 54 |
| 48 | proliferation | 54 |
| 49 | nad(+) | 52 |
| 50 | leukemia | 51 |
| 51 | age | 50 |
| 52 | children | 50 |
| 53 | zap-70 | 49 |
| 54 | in-vitro | 48 |
| 55 | disease progression | 47 |
| 56 | lymphoma | 47 |
| 57 | protein | 47 |
| 58 | transplantation | 47 |
| 59 | dysfunction | 46 |
| 60 | oxidative stress | 46 |
| 61 | cyclic adp-ribose | 45 |
| 62 | atherosclerosis | 42 |
| 63 | chemotherapy | 42 |
| 64 | mechanisms | 42 |
| 65 | minimal residual disease | 42 |
| 66 | target | 42 |
| 67 | antibody | 41 |
| 68 | breast-cancer | 41 |
| 69 | variable-region mutations | 41 |
| 70 | association | 38 |
| 71 | dendritic cells | 38 |
| 72 | markers | 38 |
| 73 | pathway | 38 |
| 74 | peripheral-blood | 38 |
| 75 | inhibition | 37 |
| 76 | microenvironment | 37 |
| 77 | gene mutation status | 36 |
| 78 | mice | 36 |
| 79 | mutations | 36 |
| 80 | progenitor cells | 36 |
| 81 | resistance | 36 |
| 82 | blood | 35 |
| 83 | efficacy | 35 |
| 84 | growth | 35 |
| 85 | in-vivo | 35 |
| 86 | microbial translocation | 35 |
| 87 | mutation status | 35 |
| 88 | nad | 35 |
| 89 | acute myeloid leukemia | 34 |
| 90 | regulatory t-cells | 34 |
| 91 | rituximab | 34 |
| 92 | classification | 33 |
| 93 | flow-cytometry | 33 |
| 94 | criteria | 32 |
| 95 | pd-1 | 32 |
| 96 | acute lymphoblastic-leukemia | 31 |
| 97 | fludarabine | 31 |
| 98 | immunity | 31 |
| 99 | individuals | 31 |
| 100 | subsets | 31 |
| 101 | aml | 30 |
| 102 | biomarkers | 30 |
| 103 | macrophages | 30 |
| 104 | t-cell-activation | 30 |
| 105 | antigen | 29 |
| 106 | induction | 28 |
| 107 | phenotype | 28 |
| 108 | plasma-cells | 28 |
| 109 | tumor microenvironment | 28 |
| 110 | antitumor-activity | 27 |
| 111 | carcinoma | 27 |
| 112 | marker | 27 |
| 113 | monoclonal-antibodies | 27 |
| 114 | mortality | 27 |
| 115 | senescence | 26 |
| 116 | autoimmunity | 25 |
| 117 | monotherapy | 25 |
| 118 | nk cells | 25 |
| 119 | stem-cell transplantation | 25 |
| 120 | down-regulation | 24 |
| 121 | memory | 24 |
| 122 | mitochondrial dysfunction | 24 |
| 123 | pathogenesis | 24 |
| 124 | cancer-cells | 23 |
| 125 | immunosenescence | 23 |
| 126 | natural-killer-cells | 23 |
| 127 | nicotinamide mononucleotide | 23 |
| 128 | population | 23 |
| 129 | b cells | 22 |
| 130 | biomarker | 22 |
| 131 | combination | 22 |
| 132 | genes | 22 |
| 133 | lymphocyte subsets | 22 |
| 134 | management | 22 |
| 135 | monocytes | 22 |
| 136 | risk-factors | 22 |
| 137 | adenosine | 21 |
| 138 | antibodies | 21 |
| 139 | impact | 21 |
| 140 | myeloma | 21 |
| 141 | t cells | 21 |
| 142 | covid-19 | 20 |
| 143 | human-immunodeficiency-virus | 20 |
| 144 | monoclonal-antibody | 20 |
| 145 | multicenter | 20 |
| 146 | outcomes | 20 |
| 147 | t-cell | 20 |
| 148 | active antiretroviral therapy | 19 |
| 149 | chronic lymphocytic leukaemia | 19 |
| 150 | cytokines | 19 |
| 151 | dna methylation | 19 |
| 152 | heterogeneity | 19 |
| 153 | immune cells | 19 |
| 154 | inhibitors | 19 |
| 155 | intima-media thickness | 19 |
| 156 | life-span | 19 |
| 157 | metastasis | 19 |
| 158 | mitochondria | 19 |
| 159 | nf-kappa-b | 19 |
| 160 | nivolumab | 19 |
| 161 | system | 19 |
